# Are natural-based isothiocyanate derivatives alternative antioxidant additives used in petrochemical products?

**DOI:** 10.1098/rsos.241929

**Published:** 2025-02-12

**Authors:** Thi Chinh Ngo, Dinh Hieu Truong, Thi Le Anh Nguyen, Quang Khuong Pham, Duy Quang Dao

**Affiliations:** ^1^ Institute of Research and Development, Duy Tan University, Da Nang 550000, Vietnam; ^2^ School of Engineering and Technology, Duy Tan University, Da Nang 550000, Vietnam; ^3^ Faculty of Pharmacy, Duy Tan University, Da Nang 550000, Vietnam

**Keywords:** isothiocyanate, DFT, FHT, RAF, antioxidant additives, petrochemical products

## Abstract

Synthetic additives used in petrochemical products, such as plastics, rubbers, resins and polymers, can enter the environment as contaminants and pollute the aquatic and soil environment. Thus, using natural-based alternative compounds has excellent potential to reduce the negative impact. In this study, the role of natural-based isothiocyanate derivatives Cp**1**–Cp**4**, i.e. allylisothiocyanate, 1-isothiocyanate-3-methylbutane, 4-methylphenyl isothiocyanate and 2-phenylethyl isothiocyanate in use as primary antioxidant additives was studied via their HO^•^-radical scavenging activity. Formal hydrogen transfer, radical adduct formation and single electron transfer mechanisms were investigated in water and pentyl ethanoate (PEA) using density functional theory at M06-2X/6-311++G(3df,3pd)//M06-2X/6-311++G(d,p) level of theory. The results illustrated the high HO^•^-scavenging activity of the isothiocyanate compounds with rate constants of about 10^8^–10^9^ M^−1^ s^−1^. Allylisothiocyanate Cp**1** represents the most efficient HO^•^-scavenger with *k*_overall_ of 5.20 × 10^9^ M^−1^ s^−1^ in water and 1.85 × 10^9^ M^−1^ s^−1^ in PEA. These results allow us to enrich the data on effective antioxidant additives from natural sources to reduce HO^•^ radicals-induced oxidation processes in different environmental conditions.

## 1. Introduction

Antioxidant additives are necessary for petrochemical products such as plastics, polymers, rubbers, fuels, lubricants and petroleum-derived paints and coatings to minimize the negative impacts of environmental oxidation on their mechanical and physicochemical properties. Synthetic primary antioxidant additives, known as radical scavengers, are frequently used in industry to protect chemical products from oxidative degradation under atmospheric conditions by neutralizing the highly reactive radicals to form stable species. These molecules can generally donate hydrogen to alkoxy radicals and alkyl peroxy radicals to produce stable hydrocarbons and alkyl hydroperoxides, thus interrupting the radical chain mechanism of the autoxidation process [1,2]. Hindered phenolics and aromatic amines, two main classes of primary antioxidants, are extensively used in automotive lubricating oils and greases [2]. Based on chemical structure and physical properties, phenolic compounds (i.e. 2,6-di-*tert*-butyl-phenol or butylated hydroxytoluene, also known as BHT, and 2,6-di-*tert*-butyl-4-methyl phenol) were commonly used [2]. Sulfur-containing hindered phenols have also been widely reported [2,3]. However, synthetic phenol, amine and organophosphite antioxidants, essential additives for preventing oxidative ageing in various industrial and consumer products, have caused contaminants and environmental health risks [4–6]. For example, *N*-(1,3-dimethylbutyl)-*N*′-phenyl-*p*-phenylenediamine (6PPD), a typical *p*-phenylenediamine antioxidant for commercial rubber products (e.g. vehicle tyres), which illustrated the effectiveness against rubber ozonation [7], has been widely detected in various dust samples as well as in human urine samples [8,9]. In addition, the ozonation reaction of 6PPD results in the formation of 6PPD-quinone, which is a very toxic compound for human health and the biosystem [7].

In green chemistry, the use of antioxidant additives from natural sources is of great interest to enhance oxidative stability and reduce toxic emissions into the environment. Hernández-Sierra *et al*. used curcumin as a natural additive in two green oils and reported its excellent ability to improve the physical properties, operational performance and oxidative stability of green oils to create effective and environmentally friendly lubricants [10]. Three natural additives—ginger, black pepper and garlic have also been studied to enhance the oxidative stability and cold flow properties of coconut oil as a bio-lubricant [11]. The antioxidant effect of natural ferulic acid and its two ester derivatives (isooctyl ferulate and ethyl ferulate) on the oxidative stability of pentaerythritol oleate (PETO) as one of the ester-based lubricants has been investigated [12]. According to the Rancimat tests, the oxidation induction time of the isooctyl ferulate/PETO mixture increased by 300% compared with the control. Recently, the potential of natural antioxidants, including *Curcuma longa* L., *Citrus aurantifolia* L. and *Eucalyptus camaldulensis* Dehn., in enhancing the oxidative and long-term storage stability of biodiesel produced from *Prosopis juliflora* oil was investigated [13]. The results reveal that adding natural antioxidants notably improved the biodiesel’s oxidative stability. This study also contributes to the extensive research on natural antioxidants and their potential to enhance biodiesel quality [13].

In this context, we are interested in evaluating the structure–reactivity relationship of different natural compounds and, thus, discovering more effective antioxidants from natural sources for application as additives in petrochemical products. Isothiocyanate (ITC) derivatives that possess an interesting substituent (i.e. the thiocyanate group, −N=C=S group) are generally found in incredible amounts in broccoli and other cruciferous vegetables, and are also well-known among many natural compounds [14]. Jang *et al*. [15] demonstrated that the antioxidant activity of extracts from broccoli (*Brassica oleracea* L.) sprouts is similar to α-tocopherol and butylated hydroxytoluene (BHT). In addition, the intrinsic antioxidant activity of nine isothiocyanate derivatives contained in this extract has been studied via their thermochemical parameters, including bond dissociation enthalpy (BDE), ionization energy and electron affinity characterized for the hydrogen atom transfer and single electron transfer (SET) mechanisms [16]. However, the kinetics of radical scavenging reactions of the ITC compounds in the previous works have not yet been elucidated. Furthermore, radical adduct formation (RAF) is also a significant radical-scavenging mechanism of several antioxidants that exhibit π-bonds [17–19], and it may compete with the other antioxidant processes. Therefore, achieving a more detailed and comprehensive study of the free radical scavenging ability of potential isothiocyanate compounds is necessary.

**Figure 1.**
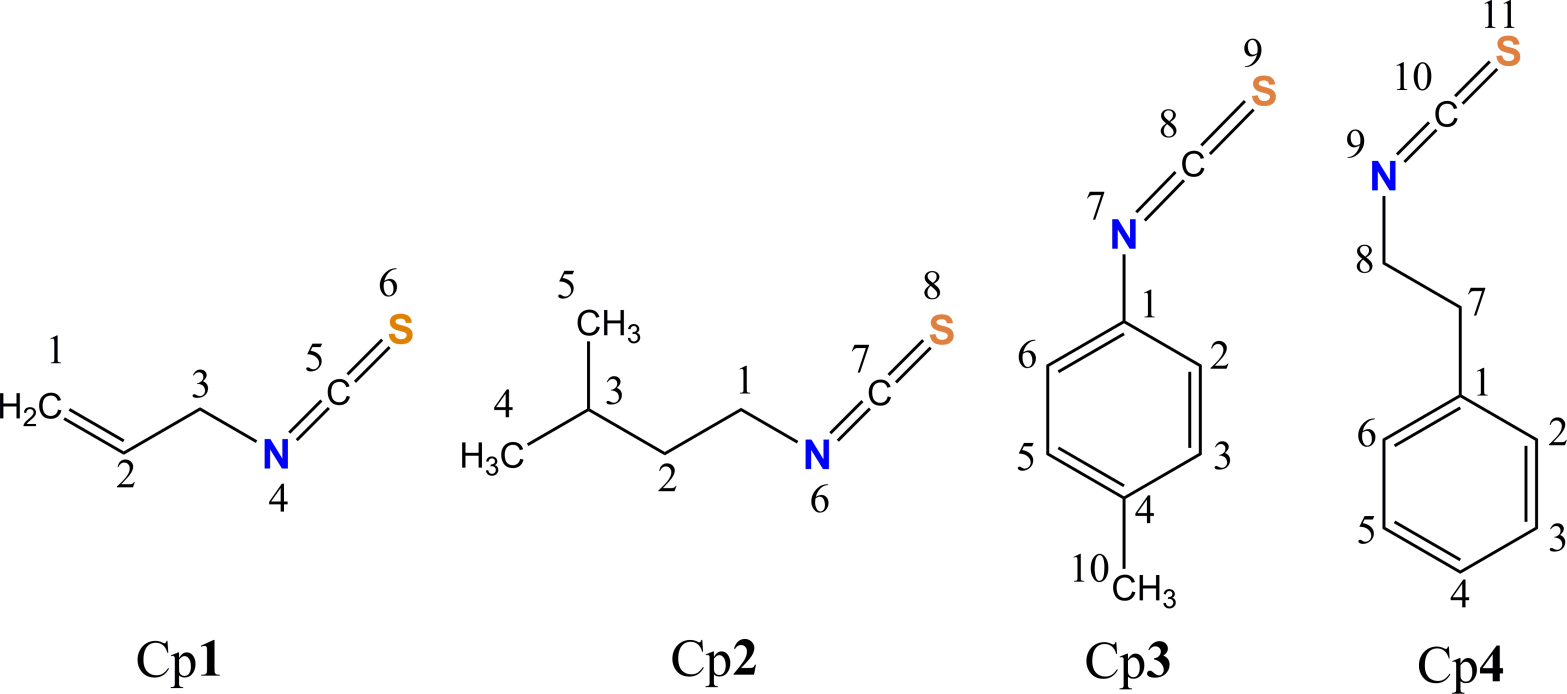
Chemical structures of the studied isothiocyanates Cp**1**–Cp**4**.

In this study, we focused on the antiradical activity of four isothiocyanate compounds, namely allylisothiocyanate (Cp**1**), 1-isothiocyanate-3-methylbutane (Cp**2**), 4-methylphenyl isothiocyanate (Cp**3**) and 2-phenylethyl isothiocyanate (Cp**4**) (figure 1). These compounds were identified in extracts of broccoli (*B. oleracea* L.) sprouts which are of interest as valuable antioxidant sources [15]. First, the thermochemical parameters, i.e. BDE, proton affinity (PA) and adiabatic ionization potential (IP), which describe intrinsic antioxidant properties, are calculated. Then, the HO^•^-scavenging reactions via three reactions, including formal hydrogen transfer (FHT), RAF and SET, are studied in two solution media with different polarity, including polar water and nonpolar pentyl ethanoate (PEA). The calculations of thermodynamic and kinetic parameters for all reactions will help to identify the efficient free radical scavengers, preferable mechanisms, favourable reaction media and the effect of functional groups on isothiocyanate structures [20,21]. Further understanding of the free radical scavenging capacity of these compounds will guide their applications as primary antioxidant additives to lubricants and plastic. Furthermore, the design of new compounds based on this exciting substituent will be particularly attractive to the petrochemical industry.

## 2. Computational methods

All calculations are performed with the Gaussian 16 Rev.C.01 package [22]. The Minnesota M06-2X [23] functional with a 6-311++G(d,p) basis set is used for structural optimization and frequency calculations. The scaling factor 0.952 [24] is applied to vibrational frequency calculations. Single point energies are then estimated at a higher level of theory, 6-311++G(3df,3pd) one. The solvation model based on the quantum mechanical charge density of a solute molecule interacting with a continuum description of the solvent (SMD) is applied for the calculations in the aqueous and PEA phases [25]. The intrinsic thermochemical parameters, i.e. BDE, PA and adiabatic IP characterized, respectively, for hydrogen transfer, proton transfer and electron transfer capacities are estimated according to the model reported in the previous studies [26–30]. Based on kinetic calculations, the quantum mechanics-based test for overall free radical scavenging activity methodology is applied to radical-molecule reactions in solution [31]. Three radical-scavenging reactions are studied as follows:

– FHT
  R−H + HO^•^ R^•^+ HOH,
– RAF
  R−H + HO^•^ → [HO−R−H]^•^, and
– SET
  R−H + HO^•^ → R−H^•+^ + HO^−^.

The enthalpy and Gibbs free energy changes of all reactions are estimated as the difference between the enthalpy and Gibbs free energy of products and that of reactants.

The kinetics of FHT and RAF reactions are calculated according to the pre-reactive complexes scheme proposed by Singleton & Cvetanovic [32]. Intrinsic reaction coordinates based on the Hessian-based predictor–corrector integrator [33,34] are also performed to verify that the imaginary frequency corresponds to the proper motion along the reaction coordinates. The Gaussian Post Processor (GPOP) program [35] is employed to calculate the rate constants of these reactions following the canonical transition state theory [36–38]:


$$k\left(T\right)=\sigma \kappa \frac{k_{B}T}{h}e^{\frac{-\Delta G^{‡}}{RT}},$$

where *σ* is the number of equivalent reaction paths and *κ* is the Eckart tunnelling factor [39]. ∆*G*^‡^ is the Gibbs free energy of activation; *T* is the temperature in Kelvin; *k*_B_ and *h* are the Boltzmann–Planck constants.

The ∆*G*^‡^ values of FHT and RAF are calculated from the difference between the transition state and reactants of these reactions, while ∆*G*^‡^ values of SET processes are estimated by Marcus’s theory [40,41]. The apparent diffusion-corrected rate constants (*k*_app_) in solution are taken into account for the thermal rate constant (*k*_T_), nearly the diffusion-limit rate constant according to the Collins–Kimball theory [31,42] and the steady-state Smoluchowski [43] rate coefficient. The Truhlar [44] and the Stokes–Einstein approaches [45,46] are also applied to calculate the mutual diffusion coefficient of the reactants. A detailed explanation is present in the electronic supplementary material.

The overall rate constants (*k*_overall_) are finally calculated as the sum of the ones of all reaction paths. Branching ratios for each reaction channel (Γ_i_, %) are also estimated by dividing its apparent rate constants (*k*_i_) by the *k*_overall_.

## 3. Results and discussion

### 3.1. Acid–base equilibria in aqueous solution

The relative pKa values are calculated in water for all studied compounds using a proton-based thermodynamic cycle [47] and methyl isothiocyanate (pKa = 12.3) as reference compounds [48]. The following equation calculates the pKa for the unknown base (HB) when the pKa of a similar acid (HA) molecule is known [47]:


$$pKa\;(HB)\;=\;pKa\;(HA)+[G_{gas}(B^{-})-G_{gas}(A^{-})-G_{gas}(HB)+G_{gas}(HA)+∆G_{sol}(B^{-})-∆G_{sol}(A^{-})-∆G_{sol}(HB)+∆G_{sol}(HA)]/RTln(10).$$

The estimated pKa values for Cp**1** to Cp**4** are 10.9, 10.8, 12.6 and 11.0, respectively. Accordingly, the neutral forms are considered for all calculations at environmental media (pH = 6–8).

### 3.2. Intrinsic thermochemical parameters characterized for the antioxidant properties

Intrinsic thermochemical parameters such as BDE, PA and IP are primary parameters to evaluate an antioxidant molecule by hydrogen, proton and electron-donating capacity, respectively. The smaller these values are, the greater donating capacities are. Although the intrinsic parameters are still far from the characterization of antioxidant properties in the solutions, it allows a rapid screening before diving deeper into the free radical scavenging activities of the studied molecules. The thermochemical parameters for the studied compounds are displayed in table 1.

**Table 1.** BDE (kcal mol^−1^), PA (kcal mol^−1^) and IP (kcal mol^−1^) of the studied compounds in water and PEA. (The bold numbers emphasize the lowest BDE and PA values representing the easiest C-H breaking bond of each compound.)

Cp	bonds	BDE		PA		IP	
		water	PEA	water	PEA	water	PEA
**Cp1**	C1−H	111.9	110.9	80.6	101.4	140.8	163.6
	C2−H	110.4	109.4	79.1	99.7		
	**C3−H**	**80.5**	**77.8**	**57.3**	**74.2**		
**Cp2**	**C1−H**	**90.5**	**88.1**	**75.1**	**92.9**	139.5	159.0
	C2−H	100.8	99.7	90.8	111.0		
	C3−H	96.3	95.8	95.2	114.4		
	C4−H	102.6	101.6	94.1	116.0		
	C5−H	101.5	100.5	94.0	114.6		
**Cp3**	C2−H	115.8	114.1	76.1	96.9	**126.8**	**145.3**
	C3−H	114.0	112.2	81.0	101.6		
	C5−H	114.6	112.2	81.0	101.6		
	C6−H	113.9	114.1	76.1	96.9		
	**C10−H**	**91.1**	**89.5**	**67.2**	**85.3**		
**Cp4**	C2−H	112.9	111.8	83.4	103.7	143.7	161.4
	C3−H	112.8	111.7	84.2	105.6		
	C4−H	113.1	112.0	84.4	106.0		
	C5−H	112.8	111.7	84.2	105.6		
	C6−H	112.4	111.8	83.4	103.7		
	**C8−H**	**90.5**	**89.7**	**73.8**	**91.4**		

It is noted that BDE values are found from 80.5 to 115.8 kcal mol^−1^ in water and from 77.8 to 114.1 kcal mol^−1^ in PEA. The lowest values obtained at C3−H (Cp**1**) owing to the electron-withdrawing effect of both C=C allylic and −N=C=S groups are comparable with the BDE of Trolox (79.8 kcal mol^−1^, water), and ascorbic acid (78.3 kcal mol^−1^, water) computed at the same level of theory. The PA changes from 57.3 to 95.2 kcal mol^−1^ (water) and from 74.2 to 116.0 kcal mol^−1^ (PEA), while larger IP values are obtained from 126.8 to 143.7 kcal mol^−1^ (water) and 145.3 to 163.6 kcal mol^−1^ (PEA). It is evident that the polar solvent lowers PA and IP values and favours the transfer processes of the charged particles like protons and electrons [27,28,49]. The most favourable positions of hydrogen and proton transfer are C3−H (Cp**1**), C1−H (Cp**2**) and C8−H (Cp**4**), generally adjacent to −N=C=S groups, except C10−H of Cp**3** at the *para*-position of the benzyl ring. Among the studied compounds, Cp**3** illustrates the most electron-donating capacity, similar to ascorbic acid (IP: 123.5 (water) and 147.1 kcal mol^−1^ (PEA)).

### 3.3. Mechanistic and kinetics evaluation of HO^•^ radical scavenging

The HO^•^-scavenging reactions of the studied compounds following three main mechanisms, i.e. FHT, RAF and SET, are discussed in this section.

#### 3.3.1. Formal hydrogen transfer reactions

**Figure 2.**
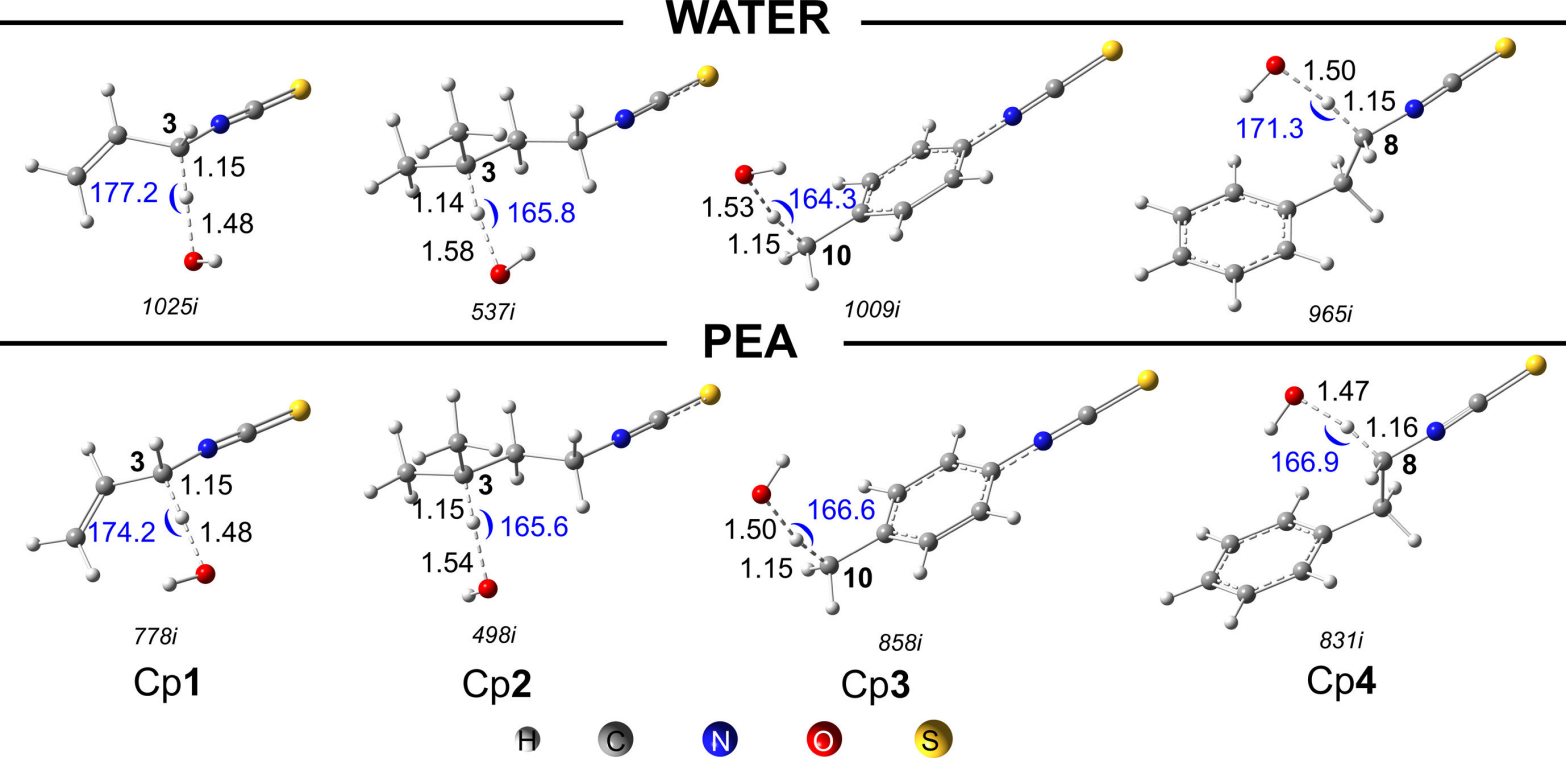
The optimized TS structures of the most favourable FHT reactions in water and PEA of Cp**1**–Cp**4**. The imaginary frequency values of each TS are given in italics. Distances of C−H and O−H are in Angstrom (Å). Angles (°) are written in blue.

**Figure 3.**
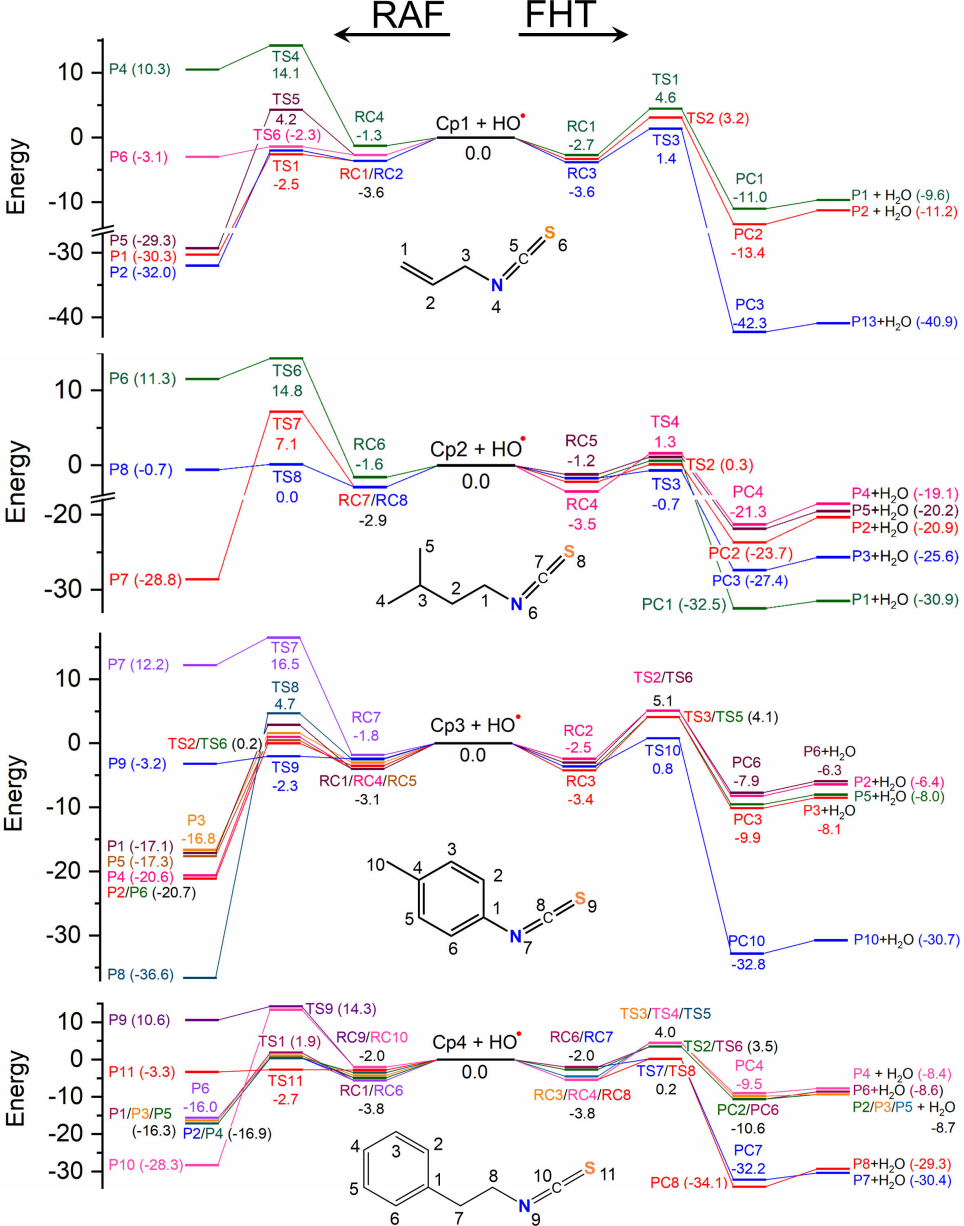
Enthalpy profile at 0 K (Δ_r_*H*_0K_, kcal mol^−1^) in water for the RAF and FHT reactions between Cp**1**–Cp**4** and HO^•^ radical. The channel numbers stand for the number of reaction sites.

FHT reactions were performed at all C−H positions of the isothiocyanates. The standard Gibbs energies of reaction (∆_r_*G*^0^), Gibbs energy of activation (∆*G*^‡^) and apparent diffusion-corrected rate constant (*k*_app_) for all FHT reactions at 298 K are summarized in table 2. The representative optimized transition states (TS) are shown in figure 2. The 0 K enthalpy profiles for these channels in water, including isolated reactants (0 kcal mol^−1^), reactant complex (RC), TS, product complex (PC) and the final product (P) are displayed in figure 3. The similar thermodynamic parameters in PEA are presented in the electronic supplementary material, table S1. Optimized structures and Cartesian coordinates of all RC, TS, PC and P are found in the electronic supplementary material, table S2. For the H-abstraction of −CH_2_− and −CH_3_ groups, a representative C−H pathway is displayed in figure 3. Similar results are obtained for other H-abstractions.

**Table 2.** Gibbs free energy of reaction (∆_r_*G*^0^, kcal mol^−1^), Gibbs free energy of activation (∆*G*^‡^, kcal mol^−1^), and apparent diffusion-corrected rate constant (*k*_app_, M^−1^ s^−1^) of FHT reactions calculated at 298 K in water and PEA. (Numbers in parentheses are the numbers of H-atoms at C-positions. *k*_app_ is the total of all H-abstraction at each C-site. Pos is the bond position. The bold text signifies the most favorable position for reaction).

Cp	Pos	water			PEA		
		∆_r_*G*^0^	∆*G*^‡^	*k*_app_	∆_r_*G*^0^	∆*G*^‡^	*k*_app_
Cp**1**	C1−(H2)	−10.4	12.4	2.03 × 10^6^	−8.5	12.6	2.29 × 10^6^
	C2−(H)	−12.0	11.6	2.37 × 10^6^	−10.3	11.6	3.80 × 10^6^
	**C3−(H2**)	−40.7	9.5	1.37 × 10^8^	−40.7	9.5	8.83 × 10^7^
	**total**			**1.42 × 10^8^**			**9.44 × 10^7^**
Cp**2**	C1−(H2)	−31.1	9.0	2.06 × 10^8^	−31.0	9.5	7.14 × 10^7^
	**C2−(H2**)	−22.1	8.3	1.00 × 10^9^	−20.3	9.0	2.17 × 10^8^
	**C3**−(**H**)	−27.2	7.2	8.56 × 10^8^	−25.3	7.2	8.18 × 10^8^
	C4−(H3)	−19.8	9.4	9.40 × 10^7^	−18.5	9.5	1.27 × 10^8^
	C5−(H3)	−20.9	9.2	2.04 × 10^8^	−19.2	9.6	1.04 × 10^8^
	**total**			**2.36 × 10^9^**			**1.34 × 10^9^**
Cp**3**	C2−(H)	−8.3	13.8	2.01 × 10^5^	−6.0	13.8	2.11 × 10^5^
	C3−(H)	−8.9	12.0	2.06 × 10^6^	−7.2	12.9	5.13 × 10^5^
	C5−(H)	−8.6	12.0	1.91 × 10^6^	−7.4	12.9	5.19 × 10^5^
	C6−(H)	−7.0	13.8	2.06 × 10^5^	−5.3	12.6	1.69 × 10^6^
	**C10**−(**H3**)	−30.0	8.6	7.28 × 10^8^	−28.5	8.9	3.77 × 10^8^
	**total**			**7.32 × 10^8^**			**3.80 × 10^8^**
Cp**4**	C2−(H)	−9.9	12.6	5.14 × 10^5^	−7.9	12.2	1.21 × 10^6^
	C3−(H)	−9.2	11.7	2.20 × 10^6^	−7.7	11.7	3.05 × 10^6^
	C4−(H)	−8.9	11.7	2.19×10^6^	−7.3	12.5	9.35 × 10^5^
	C5−(H)	−9.2	11.7	2.13×10^6^	−7.7	11.7	3.01 × 10^6^
	C6−(H)	−8.0	12.6	5.09 × 10^5^	−7.9	12.2	1.30 × 10^6^
	C7−(H2)	−29.3	8.7	3.25 × 10^8^	−29.2	9.3	1.11 × 10^8^
	**C8**−(**H2**)	−28.8	8.5	5.57 × 10^8^	−30.2	8.9	2.74 × 10^8^
	**total**			**8.90 × 10^8^**			**3.94 × 10^8^**

Figure 2 illustrates the optimized structures of the TS for the most favourable FHT reactions in both water and PEA. The distances between the H-atom and O-atom of the HO***^•^*** radical are 1.48−1.58 Å (water) and 1.47−1.54 Å (PEA), while the C−H distances are shorter, being 1.14−1.15 Å (water), and 1.15−1.16 Å (PEA). The C−H−O angles vary from 164.3^o^ to 177.2^o^.

The reaction enthalpies at 0 K (∆_r_*H*_0K_) of the FHT reactions are primarily negative, −40.9 to −6.3 kcal mol^−1^ (water) (figure 3), and −40.8 to −4.7 kcal mol^−1^ (PEA) (electronic supplementary material, table S1), that are assigned to spontaneous and exothermic reactions. The most thermodynamically favourable FHT reactions of Cp**1** are found in both media at the easiest breaking C3−H bond, which is consistent with the electron-withdrawing effect of both C1=C2 and N4=C5=S6 groups (figure 1). Similarly, the most thermodynamically favourable FHT reactions of other isothiocyanates are at C1−H (for Cp**2**), C10−H (for Cp**3**) and C7/C8−H (for Cp**4**) (figure 3) that is also related to the electron-withdrawing of the isothiocyanate group or benzene ring. This observation also agrees with the previous work on sulforaphane, where H-abstraction is most favourable at the α-C of the isothiocyanate group [50]. Accordingly, the enthalpies of the TS for those reactions are also relatively low, being 1.4 (C3−H, Cp**1**), 0.4 (C1−H, Cp**2**), 0.8 (C10−H, Cp**3**) and 0.2 (C7/C8−H, Cp**4**) kcal mol^−1^.

The Gibbs free energies of reaction (∆_r_*G*^0^) at 298 K show relatively negative values from −40.7 to −7.0 kcal mol^−1^ (water) and −40.7 to −5.3 kcal mol^−1^ (PEA) (table 2). The activation energy ∆*G*^‡^ values vary from 7.2 to 13.8 (water) and from 7.2 to 13.8 kcal mol^−1^ (PEA). It should be noted that the H-abstraction of aliphatic C−H is more exothermic and has lower barriers than that of aromatic C−H, consistent with the previous calculation results [51]. Regarding the kinetics, the thermal rate constants (*k*_T_) and diffusion rate constants (*k*_D_) of these reactions are presented in the electronic supplementary material, table S3. The calculated *k*_D_ values are generally found around 10^9^ M^−1^ s^−1^. Here, we discuss the apparent diffusion-corrected rate constants (*k*_app_) to identify efficient reactions. The results in table 2 demonstrate that the highest *k*_app_ values of each compound are obtained in water; for example, *k*_app_ = 1.37 × 10^8^ (C3−H, Cp**1**), 8.56 × 10^8^ (C3−H, Cp**2**), 7.28 × 10^8^ (C10−(H3), Cp**3**) and 5.57 × 10^8^ M^−1^ s^−1^ (C8−H, Cp**4**). The total FHT rates in water are sorted in the following order: 2.36 × 10^9^ (Cp**2**) > 8.90 × 10^8^ (Cp**4**) > 7.32 × 10^8^ (Cp**3**) > 1.42 × 10^8^ M^−1^s^−1^ (Cp**1**), while the reaction rates in PEA are lower but also in the same order (table 2). The highest FHT rates of Cp**2** could be explained by the higher number of methyl and methylene groups than the unsaturated –C=C−H and aromatic =C−H of the benzene ring.

#### 3.3.2. Radical adduct formation reactions

**Figure 4.**
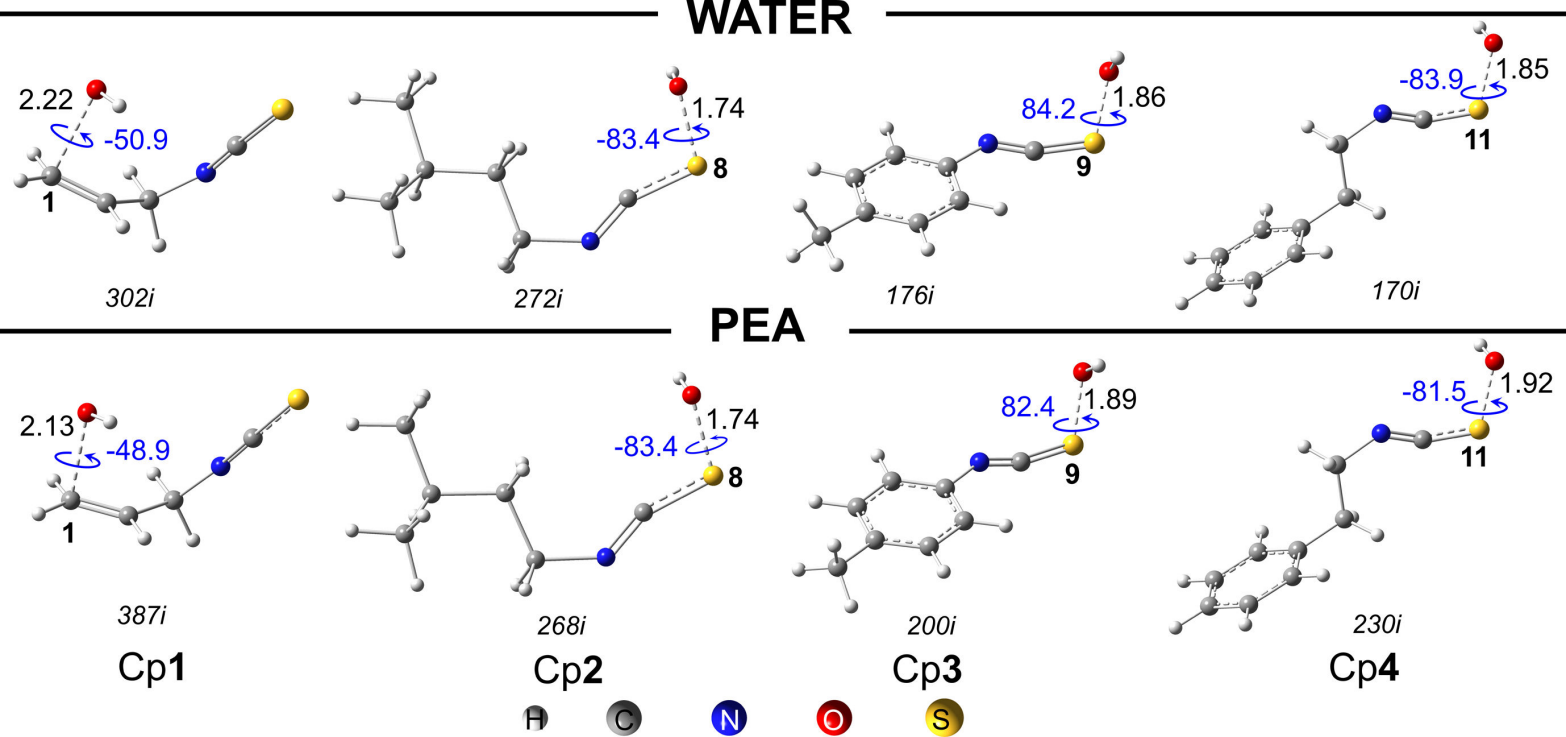
The optimized TS structures of the most favourable RAF reactions in water and PEA of Cp**1**–Cp**4**. The imaginary frequency values of each TS are given in italics. Distances of C−H and O−H are in Angstrom (Å). Angles (°) are written in blue.

The RAF reactions are performed at all potential atomic sites, i.e. C, N and S of unsaturated bonds of the studied molecules. The energy profiles at 0 K in water, containing isolated reactants at 0 kcal mol^−1^, RC, TS and P are described in figure 3. Other results calculated in PEA are given in the electronic supplementary material, table S4. The standard Gibbs energies of reaction (∆_r_*G*^0^), Gibbs energy of activation (∆*G*^‡^) and apparent rate constant (*k*_app_) at 298 K for all RAF reactions are shown in table 2. Optimized structures and Cartesian coordinates of RC, TS and P are presented in the electronic supplementary material, table S5. The optimized TS structures for the most favourable reactions of each compound are displayed in figure 4.

For Cp**1**, the HO^•^-addition occurs at the C1-position, while the addition of Cp**2**, Cp**3** and Cp**4** is more favourable at the S-positions. It is observed that the C−O distances of the reaction site and the C=C−O−H dihedral values are slightly varied in the two media. For example, the C−O distances of the reaction site for Cp**1** are 2.22 Å (water) and 2.13 Å (PEA), and the C=C−O−H dihedral values are −50.9 (water) and −48.9° (PEA).

The results obtained at 0 K showed that exothermic reactions are detected for the HO^•^-addition to all C-atoms of the studied compounds, with relatively negative enthalpies from −36.6 to −16.0 kcal mol^−1^ (water) and from −34.6 to −14.7 kcal mol^−1^ (PEA) (figure 3). The obtained values for the RAF at S positions are less negative, being from −3.3 to −0.7 kcal mol^−1^ (water) and from −2.6 to 0.0 kcal mol^−1^ (PEA). By contrast, ∆_r_*H*_0K_ values of reaction at N are positive, i.e. from 10.3 to 12.2 kcal mol^−1^ (water) and 9.1 to 12.1 kcal mol^−1^ (PEA), corresponding to the endothermic processes.

Regarding the results obtained at 298 K, the ∆_r_*G*^0^ of C-addition reactions showed negative values in both phases, from −26.7 to −6.7 kcal mol^−1^ (water) and −23.8 to −5.9 kcal mol^−1^ (PEA). By contrast, positive values are observed for N and S additions, i.e. from 19.2 to 22.1 kcal mol^−1^ (water), from 18.3 to 21.7 kcal mol^−1^ (PEA) (at N) and from 4.8 to 8.5 kcal mol^−1^ (water) and 5.3 to 8.3 kcal mol^−1^ (PEA) (at S), respectively (table 2). Generally, the ∆*G*^‡^ values for the C-addition reactions are found from 6.2 to 22.3 kcal mol^−1^ (water) and from 7.1 to 23.2 kcal mol^−1^ (PEA). On the other hand, the ∆*G*^‡^ of N-addition reactions in water varies from 23.3 to 25.8 kcal mol^−1^ (water) and 22.3–25.4 kcal mol^−1^ (PEA), whereas lower values of 6.2–9.2 kcal mol^−1^ (water), and 6.6–9.7 kcal mol^−1^ (PEA) are obtained for the S additions (table 3).

**Table 3.** Gibbs free energy of reaction (**∆**_r_*G*^0^, kcal mol^−1^), Gibbs free energy of activation (∆*G*^‡^, kcal mol^−1^) and apparent diffusion-corrected rate constant (*k*_app_, M^−1^ s^−1^) of RAF reactions calculated at 298 K in water and PEA. (Pos is the bond position. The bold text signifies the most favorable position for reaction).

Cp	Pos	water			PEA		
		∆_r_*G*^0^	∆*G*^‡^	*k*_app_	∆_r_*G*^0^	∆*G*^‡^	*k*_app_
Cp**1**	**C1**	−21.1	6.2	1.83 × 10^9^	−21.2	7.1	9.87 × 10^8^
	C2	−23.1	6.4	1.69 × 10^9^	−22.0	7.7	4.75 × 10^8^
	C5	−19.4	13.2	6.14 × 10^4^	−16.2	13.9	1.91 × 10^4^
	N4	19.8	23.4	2.29 × 10^–3^	18.3	22.6	9.26 × 10^–3^
	S6	5.5	6.4	1.54 × 10^9^	6.3	8.0	3.02 × 10^8^
	**total**			**5.05 × 10^9^**			**1.76 × 10^9^**
Cp**2**	C7	−19.0	15.9	5.81 × 10^2^	−18.9	17.2	6.34 × 10^1^
	N6	20.9	23.3	3.18 × 10^–3^	18.9	22.9	5.54 × 10^–3^
	**S8**	8.5	9.2	4.72 × 10^7^	8.3	9.8	1.74 × 10^7^
	total			**4.72 × 10^7^**			**1.74 × 10^7^**
Cp**3**	C1	−8.5	11.2	1.57 × 10^6^	−6.0	13.9	1.88 × 10^4^
	C2	−11.1	8.8	9.29 × 10^7^	−10.3	10.8	3.31 × 10^6^
	C3	−7.9	10.1	1.05 × 10^7^	−6.1	11.3	1.50 × 10^6^
	C4	−11.0	10.1	1.02 × 10^7^	−10.7	11.3	1.50 × 10^6^
	C5	−8.2	9.1	5.42 × 10^7^	−7.2	11.3	1.51 × 10^6^
	C6	−11.1	8.8	9.07 × 10^7^	−10.3	10.8	3.30 × 10^6^
	C8	−26.7	13.4	4.62 × 10^4^	−23.7	14.8	4.40 × 10^3^
	N7	22.1	25.8	4.49 × 10^–5^	21.7	25.4	7.94 × 10^–5^
	**S9**	5.7	6.5	1.41 × 10^9^	5.3	6.6	1.34 × 10^9^
	**total**			**1.67 × 10^9^**			**1.35 × 10^9^**
Cp**4**	C1	−6.7	11.1	1.85 × 10^6^	−6.5	12.5	2.06 × 10^5^
	C2	−7.6	9.2	4.65 × 10^7^	−6.9	9.4	3.69 × 10^7^
	C3	−7.3	9.6	2.56 × 10^7^	−5.6	11.5	1.10 × 10^6^
	C4	−8.5	8.1	2.70 × 10^8^	−7.2	11.0	2.59 × 10^6^
	C5	−7.3	9.6	2.59 × 10^7^	−5.9	11.5	1.10 × 10^6^
	C6	−7.0	8.8	9.11 × 10^7^	−7.2	10.6	4.41 × 10^6^
	C10	−18.1	22.3	1.89 × 10^–2^	−16.1	23.8	1.45 × 10^–3^
	N9	19.2	23.4	2.54 × 10^–3^	18.6	22.3	1.47 × 10^–2^
	**S11**	4.8	6.2	1.66 × 10^9^	6.5	7.9	3.33 × 10^8^
	**total**			**2.12 × 10^9^**			**3.79 × 10^8^**

Moreover, the kinetic calculations based on thermodynamic results are also discussed. Similar to FHT reactions, the calculated *k*_D_ values of RAF are found in the 10^9^ M^−1^ s^−1^ range. The thermal and diffusion rate constants of all RAF reactions are summarized in the electronic supplementary material, table S6.

The results show that the highest apparent rate constant of the RAF reactions is at the C1=C2 allylic site of Cp**1**, i.e. *k*_app_ in water being 1.83 × 10^9^ and 1.69 × 10^9^ M^−1^ s^−1^ for C1 and C2, respectively (table 3). The results also suggest that electrophilic free radical addition to allylic C is higher than other aromatic C of benzene rings and the C of the isothiocyanate group. In the second place, with a free electron pair on the S-atoms of the isothiocyanate groups, adding electrophilic agents like hydroxyl radicals to these atoms is favourable [52,53]. The high rate constants are also obtained for these RAF reactions, i.e. 1.54 × 10^9^ (Cp**1**), 4.72 × 10^7^ (Cp**2**), 1.41 × 10^9^ (Cp**3**) and 1.66 × 10^9^ M^−1^ s^−1^ (Cp**4**). By contrast, the rate constants of N-addition reactions are relatively low (table 3). It is worth noting that the HO^•^-addition to an S-atom on the −N=C=S group attached to an alkyl chain (Cp**2**) is weaker than that of the ones bonded to electron-withdrawing substituents of the other compounds [14,52].

**Figure 5.**
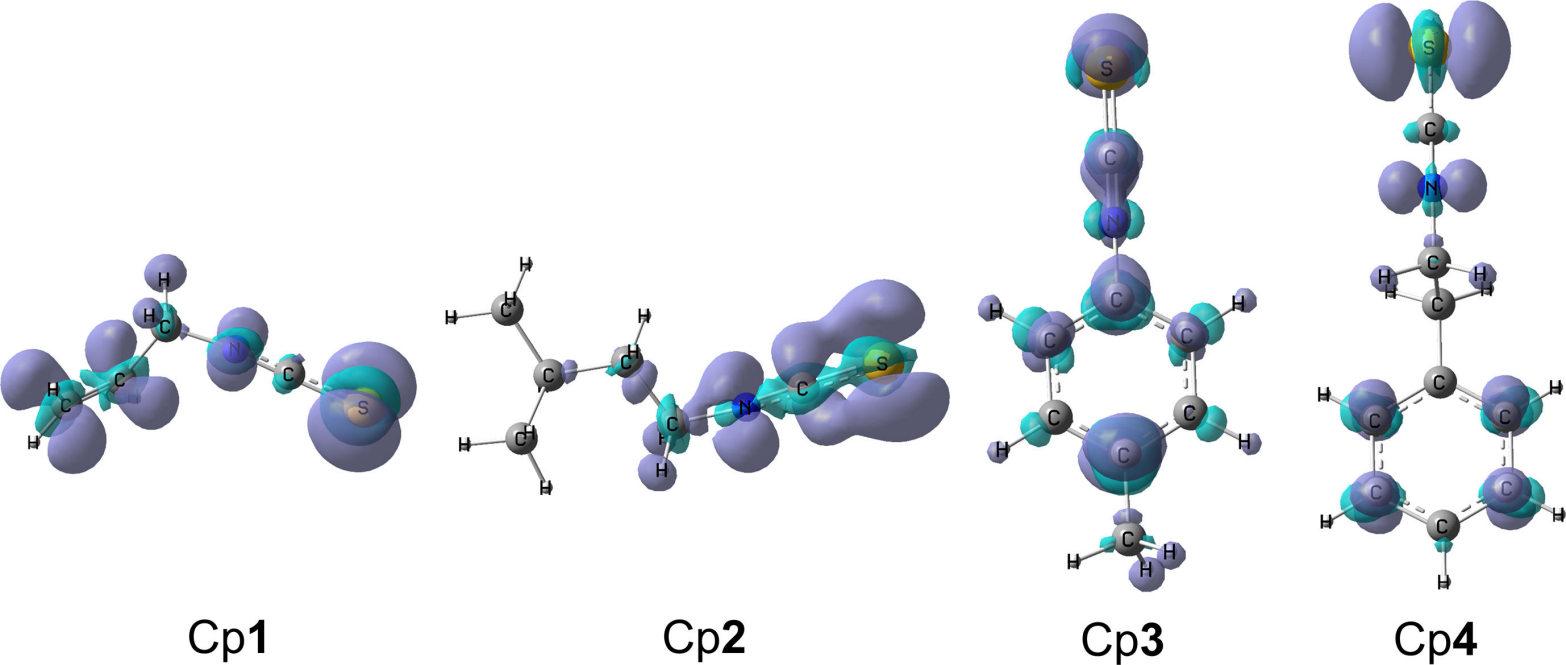
Fukui function plot for radical attack (*f *^0^) (iso-value = 0.003) describing the favourable reactive sites of Cp**1**–Cp**4** calculated in water.

The HO^•^-scavenging ability of these isothiocyanates can be explained by the local chemical quantum indices, namely the Fukui function for radical attack (*f *^0^) proposed by Yang *et al*. [54–56] which allows us to identify favourable reaction sites for the radical attack on a molecule. The *f *^0^ was calculated using a multifunctional program for wave function analysis (Multiwfn) [57]. As a result, the larger *f *^0^ densities are found at the –N=C=S group of all the studied molecules, confirming the essential role of the isothiocyanate group in the radical scavenging activities. Furthermore, the C1=C2 allylic group of the Cp**1** also displays a significant density for the  *f *^0^ function in comparison with the other functional groups of Cp**2**, Cp**3** and Cp**4** compounds (figure 5). This observation confirms the higher rate constant of the Cp**1** than the other ones.

In addition, the total RAF rate constants of Cp**1** are the highest in both phases, with values of 5.05 × 10^9^ M^−1^ s^−1^ (water) and 1.76 × 10^9^ M^−1^ s^−1^ (PEA). These rate constants are close to the diffusion-controlled rate owing to high values of both C-addition (at C1=C2 allylic site) and S-addition reactions. By contrast, having a saturated carbon chain, additional reactions of Cp**2** only occur at the C- and S-positions of the isothiocyanate group, resulting in the smallest rate constant. The C-additions on the benzene ring contribute to RAF rate constants of Cp**3** and Cp**4**. Following the Cp**1**, the total RAF rate constants are 2.12 × 10^9^ (Cp**4**), 1.67 × 10^9^ (Cp**3**), 4.72 × 10^7^ M^−1^s^−1^ (Cp**2**) in water and 1.35 × 10^9^ (Cp**3**), 3.79 × 10^8^ (Cp**4**) and 1.74 × 10^7^ M^−1^ s^−1^ (Cp**2**) in PEA. Thus, RAF reactions more preferably occur in water than in PEA.

#### 3.3.3. Single electron transfer reactions

The SET reaction simulates the capacity of a compound to yield an electron to HO^•^ radicals. The Gibbs free energy of reaction (∆_r_*G*^0^), Gibbs free energy of activation (∆*G*^‡^) and rate constant (*k*_SET_) for SET reactions are displayed in table 4.

**Table 4.** Gibbs free energy of reaction (**∆**_r_*G*^0^, kcal mol^−1^), Gibbs free energy of activation (∆*G*^‡^, kcal mol^−1^), and rate constant (*k*_SET_, M^−1^ s^−1^) of SET reactions calculated at 298 K in water and PEA.

Cp	water			PEA		
	*Δ*_r_ *G* ^0^	*∆G* ^‡^	*k*_SET_	*Δ*_r_ *G* ^0^	∆*G*^‡^	*k*_SET_
Cp**1**	30.0	106.8	7.90 × 10^–65^	78.7	106.9	4.15 × 10^–79^
Cp**2**	28.6	57.3	4.46 × 10^–28^	73.9	259.5	8.95 × 10^–177^
Cp**3**	15.4	20.7	9.65 × 10^–2^	60.0	190.8	1.99 × 10^–126^
Cp**4**	31.7	122.3	3.18 × 10^–66^	75.9	183.1	7.53 × 10^–121^

As observed in table 4, the ∆_r_*G*^0^ of SET reactions are obtained from 15.4 to 31.7 kcal mol^−1^ in water and greater values from 60.0 to 78.7 kcal mol^−1^ in PEA, which denotes the endothermic processes. Moreover, the ∆*G*^‡^ values are also high, from 20.7 to 122.3 kcal mol^−1^ (water) and 106.9–259.5 kcal mol^−1^ (PEA). Thus, the SET reactions of all studied compounds are less favourable than FHT and RAF reactions, with very low rate constants in both phases (table 4). This could be explained by their low electron-donating ability, i.e. the calculated ionization energy in water being 140.8, 139.5, 126.8 and 143.7 kcal mol^−1^, from Cp**1** to Cp**4**, respectively. However, owing to the favourable electron transfer process in polar solvents, the SET process in water is more favourable than in PEA.

Finally, the overall apparent rate constants (*k*_overall_) of all studied compounds and the contribution of each reaction mechanism are summarized in table 5.

**Table 5.** Overall apparent rate constants (*k*_overall,_ M^−1^ s^−1^) and branching ratios (Γ_i_, %) for FHT, RAF and SET reactions of Cp**1**–Cp**4** with HOꞏ radical in water and PEA.

Cp	*k*_overall_	*k*_FHT_	*k*_RAF_	*k*_SET_	Γ_FHT_	Γ_RAF_	Γ_SET_
**water**							
Cp**1**	5.20 × 10^9^	1.42 × 10^8^	5.05 × 10^9^	7.90 × 10^–65^	2.7	97.3	0.0
Cp**2**	2.41 × 10^9^	2.36 × 10^9^	4.72 × 10^7^	4.46 × 10^–28^	98.0	2.0	0.0
Cp**3**	2.40 × 10^9^	7.32 × 10^8^	1.67 × 10^9^	9.65 × 10^–2^	30.5	69.5	0.0
Cp**4**	3.01 × 10^9^	8.90 × 10^8^	2.12 × 10^9^	3.18 × 10^–66^	29.5	70.5	0.0
**PEA**							
Cp**1**	1.85 × 10^9^	8.36 × 10^7^	1.76 × 10^9^	4.15 × 10^–79^	4.5	95.5	0.0
Cp**2**	1.35 × 10^9^	1.34 × 10^9^	1.74 × 10^7^	8.95 × 10^–177^	98.7	1.3	0.0
Cp**3**	1.73 × 10^9^	3.80 × 10^8^	1.35 × 10^9^	1.99 × 10^–126^	22.0	78.0	0.0
Cp**4**	7.74 × 10^8^	3.94 × 10^8^	3.79 × 10^8^	7.53 × 10^–121^	51.0	49.0	0.0

First, it is interesting to note that Cp**1** is the most effective HO^•^ scavenger in both phases, showing the most important rate constant, i.e. 5.20 × 10^9^ (water) and 1.85 × 10^9^ (PEA) (table 5). This observation is related to the high rate constant of RAF reactions at the C1=C2 allylic and the S site. The interaction of Cp**1** with the HO^•^ radical is slightly lower than that of Trolox, a standard antioxidant, with rate constants of 2.78 × 10^10^ in water and 1.47 × 10^10^ in PEA [58]. The scavenging activity of the isothiocyanates is arranged as follows: Cp**1** > Cp**4** > Cp**2** ~ Cp**3** (water) and Cp**1** > Cp**3** > Cp**2** > Cp**4** (PEA). Second, RAF reaction is predominant for Cp**1** (*Γ*_RAF_ ~ 97.3%), Cp**3** and Cp**4** (*Γ*_RAF_~70%), owing to a significant contribution of the addition at the S-atom of the isothiocyanate group, while FHT at the aliphatic chain is more critical for Cp**2** (*Γ*_FHT_~98.0%) in both phases. The SET reaction does not contribute to the HO^•^-scavenging process. In conclusion, the additions at S sites largely contribute to the reactions with the HO^•^ radical that confirms the crucial role of the –N=C=S group in the antiradical activity.

Finally, the rate constants of HO^•^- isothiocyanates reactions in aqueous solution (2.40 × 10^9^–5.20 × 10^9^ M^−1^ s^−1^) are compared with that of HO^•^ with poly(vinyl alcohol) and its low-molecular-weight model compound pentane-2,4-diol being 1.50 × 10^8^ and 2.30 × 10^9^ M^−1^ s^−1^, respectively [59]. Moreover, the highest rate constant of Cp**1** (5.20 × 10^9^ M^−1^ s^−1^) is similar to the rate for the reactions of HO^•^ radicals with several monomers (as shown in table 6) [60].

**Table 6.** The rate constants (*k_i_*, M^−1^ s^−1^) for the reactions of HO^•^ radicals with several monomers (pH = 7.0–7.2) [60].

monomers	acrylic acid	acrylamide	acrylonitrile	crotonic acid	allyl alcohol
***k_i_***	5.74 × 10^9^	4.74 × 10^9^	5.18 × 10^9^	4.91 × 10^9^	5.92 × 10^9^

Thus, this comparison suggests that the studied compounds may serve as potential antioxidant additives that react more quickly with the reactive free radicals available in the aquatic environment, slowing down the degradation process of the protected polymers or plastics. Our finding of the antiradical scavenging activity of ITC compounds would also provide insightful information for designing the novel antioxidant molecules with –N=C=S structural framework conjugated with π-systems. These promising compounds will have potential applications as effective antioxidant additives for oxidative protection in the industry.

## 4. Conclusion

This work employs the Density Functional Theory approach to effectively study the antiradical mechanisms and kinetics of the isothiocyanate compounds. The results demonstrated that all studied compounds exhibit high HO^•^-scavenging activity, with high rate constants of about 10^8^–10^9^ M^−1^ s^−1^. First, the Cp**1** represents the most efficient HO^•^-scavenger in both water (*k*_overall_: 5.20 × 10^9^ M^−1^ s^−1^) and PEA (*k*_overall_: 1.85 × 10^9^ M^−1^ s^−1^), owing to the high rate constants of RAF reactions at the allylic and S sites. Notably, the isothiocyanate group largely contributes to the antiradical activity of these compounds. While the RAF reactions are prominent in Cp**1** (97.3 %), Cp**3** (69.5 %) and Cp**4** (70.5 %) owing to the presence of several nonsaturated carbons (C=C allylic, aromatic and isothiocyanate), the FHT is more critical for Cp**2** (98.0%) owing to the aliphatic chain. Second, the HO^•^-scavenging reactions in water are more favourable than in PEA, showing an apparent solvent effect. Finally, the high rate constants of HO^•^-scavenging reactions of the studied compounds suggest their role as primary antioxidant additives to minimize the oxidative degradation caused by the highly reactive radicals available in the environments. The promising natural additives with high antioxidant capacity will also be environmentally friendly.

## Data Availability

All relevant necessary data to reproduce all results in the paper are within the main text. Electronic supporting information (ESI) files: Dryad [61]. Supplementary material is available online [62].
